# Supplementary material for: Associations between Fecal Contamination of the Household Environment and Enteric Pathogen Detection in Children Living in Maputo, Mozambique
Source: Environ Health (Wash). 2025 Apr 11;3(7):757–67. doi: 10.1021/envhealth.4c00283 (PMC12281200; doi:10.1021/envhealth.4c00283)
Supplement: Supplementary file 1 [file eh4c00283_si_001.pdf]

Supporting Information: Associations between fecal contamination of the household environment and enteric pathogen detection in children living in Maputo, Mozambique

David A. Holcomb[1], Jackie Knee[2], Zaida Adriano[3], Drew Capone[4], Oliver Cumming[2], Erin Kowalsky[1], Rassul Nalá[5], Edna Viegas[6], Jill R. Stewart[1,7], Joe Brown[1,*]

1. Department of Environmental Sciences and Engineering, Gillings School of Global Public Health, University of North Carolina at Chapel Hill, Chapel Hill, North Carolina 27599, United States of America
2. Department of Disease Control, Faculty of Infectious and Tropical Diseases, London School of Hygiene and Tropical Medicine, London WC1E 7HT, United Kingdom
3. WE Consult ltd, Maputo, Mozambique
4. Department of Environmental and Occupational Health, School of Public Health, Indiana University, Bloomington, Indiana 47405, United States of America
5. Division of Parasitology, Instituto Nacional de Saúde, Marracuene, Mozambique
6. Centro de Investigação e Treino em Saúde da Polana Caniço, Instituto Nacional de Saúde, Maputo, Mozambique
7. Baruch Institute for Marine and Coastal Sciences, School of the Earth, Ocean and Environment, University of South Carolina, Georgetown, South Carolina 29442, United States of America

* Corresponding Author: Department of Environmental Sciences and Engineering, University of North Carolina, CB #7431, Chapel Hill, North Carolina 27599-7431, USA; joebrown@unc.edu; 919-962-2756

This supplement contains 27 pages, 3 figures, and 8 tables.

Table of Contents

S1 Stool Processing and Molecular Analysis ... 3

S2 Environmental Sample Molecular Analysis ... 4

S3 Molecular Fecal Marker Quantification ... 7

S4 Detection Limits ... 8

S5 Laboratory Quality Control ... 9

S6 Multiple Pathogen Pooled Regression Model ... 10

S7 Prior Distributions ... 12

S8 Posterior Predicted Probabilities for Continuous Exposures ... 14

S9 Fecal Marker Occurrence by Outcome Status ... 15

S10 Marginal Prevalence Estimates ... 17

S11 Supplementary References ... 25

S1 Stool Processing and Molecular Analysis

Stool collection materials supplied to the caregivers of enrolled children included diapers and pre-labeled sterile sample bags. For older children who no longer wore diapers, we also provided a plastic potty and requested that the caregiver transfer stool collected in the potty to the provided diaper and place the diaper in the sterile sample bag for retrieval. When a stool sample was too liquid to aliquot directly into storage tubes, we instead stored a piece of the soaked diaper material in a 2 mL cryovial. Microscopy was not performed on the liquid stool samples. We isolated nucleic acids from bulk stool following the GPP manufacturer’s protocol, pretreating bulk stool samples with 1 mL of ASL stool lysis buffer (Qiagen, Hilden, Germany). We eluted diaper samples in 2.5 mL of ASL buffer using a sterile 10 mL syringe to forcefully rinse the sample five times and eluted rectal swabs by agitating in 1 mL of lysis buffer for 1 min. Nucleic acids were isolated from 1 mL of pretreated stool or eluate using the QIAamp 96 Virus QIAcube HT Kit on the QIAcube HT platform (Qiagen).[1] We added the nonpathogenic RNA virus MS2 to the pretreated samples immediately before performing the extractions as a specimen processing control (SPC) to assess extraction efficiency and RT-PCR inhibition. Each set of extractions included at least one negative extraction control (NEC) containing only lysis buffer and MS2, as well as an extraction blank with lysis buffer only. Stool extracts were stored at 4 °C and analyzed by GPP within 24 hours. For approximately 10% of samples, we extracted and analyzed a second aliquot as a biological replicate. All GPP analyses included a no-template control (NTC) containing only molecular-grade water and PCR reagents.

S2 Environmental Sample Molecular Analysis

We isolated DNA from soil sample filters using the DNeasy PowerSoil Kit (Qiagen) and from water sample filters with the DNA-EZ ST01 Kit (GeneRite, North Brunswick, NJ, USA).[2] Each extraction batch of up to 22 sample filters included both a positive control (PC), consisting of a clean filter spiked with 2×10^8 copies of composite DNA standard reference material (Table S1), and an NEC with a clean filter only.[3] We added 3 µg salmon testes DNA (Sigma-Aldrich, St. Louis, MO, USA) to each lysis tube (including PC and NEC tubes) as a specimen processing control.[4]

Table S1. Synthetic DNA reference material spiked into positive controls

**assays covered**	**sequence [5'-3']**	**GenBank (base positions)**	**length [bases]**
BacHum-UCD[5]<br>BacUni-UCD[5]<br>HF183/BacR287[6]	CCAGGATGGGATCATGAGTTCACATGTCCGCATGATTAA<br>AGGTATTTTCCGGTAGACGATGGGGATGCGTTCCATTAG<br>ATAGTAGGCGGGGTAACGGCCCACCTAGTCAACGATGGA<br>TAGGGGTTCTGAGAGGAAGGTCCCCCACATTGGAACTGA<br>GACACGGTCCAAACTCCTACGGGAGGCAGCAGTGAGGA<br>ATATTGGTCAATGGGCGATGGCCTGAACCAGCCAAGTAG<br>CGTGAAGGATGACTGCCCTATGGGTTGTAAACTTCTTTTA<br>TAAAGGAATAAAGTCGGGTATGCATACCCGTTTGCATGT<br>ACTTTATGAATAAGGATCGGCTAACTCCGTGCCAGCAGC<br>CGCGGTAATACGGAGGATCCGAGCGTTATCCGGATTTAT<br>TGGGTTTAAAGGGAGCGTAGATGGATGTTTAAGTCAGTT<br>GTGAAAGTTTGCGGCTCAACCGTAAAATTGCAGTTGATA<br>CTGGATGTCTTGAGTGCAGTTGAGGCAGGCGGAATTCGT<br>GGTGTAGCGGTGAAATGCTTAGATATCACGAAGAACTCC<br>GATTGCGAAGGCAGC	AB242142<br>(170-730)	560
GFD[7]<br>LA35[8]	TGGGTCTAATACCGGATACGACCATCTGCCGCATGGCGG<br>GTGGTGGAAAGTTTTTCGATTGGGGATGGGCTCGCGGCC<br>TATCAGTTTGTTGGTGGGGTAATGGCCTACCAAGGCGAC<br>GACGGGTAGCCGGCCTGAGAGGGCGACCGGCCACACTG<br>GGACTGAGACACGGCCCAGACTCCTACGGGAGGCAGCA<br>GTGGGGAATATTGCACAATGGGGGAAACCCTGATGCAGC<br>GACGCAGCGTGCGGGATGACGGCCTTCGGGTTGTAAACC<br>GCTTTCAGCAGGGAAGAAGCCTTCGGGTGACGGTACCTG<br>CAGAAGAAGTACCGGCTAACTACGTGCCAGCAGCCGCG<br>GTAATACGTAGGGTACGAGCGTTGTCCGGAATTATTGGG<br>CGTAAAGAGCTCGTAGGTGGTTGGTCACGTCTGCTGTGG<br>AAACGCAACGCTTAACGTTGCGCGGGCAGTGGGTACGGG<br>CTGACTAGAGTGCAGTAGGGGAGTCTGGAATTCCTGGTG<br>TAGCGGTGAAATGCGCAGATATCAGGAGGAACACCGGT<br>GGCGAAGGCGGGACTCTGGGCTGTGACTGACACTGGGG<br>AGCGAAAGCATTGCTAACAGTTcGGCTGAGCACTCTAGG<br>GAGACTGCCTTCGCAAGGAGGAGGAAGGTGAGGACGAC<br>GTCAAGTCATCATGGCCCTTACGCCTAGGGCTACACACG<br>TGCTACAATGGGATGTACAAAGAGACGCAATACCGCGA	FJ462358 (156-<br>746)<br>JN084061<br>(29-171)	732
EC23S857[9]<br>HAdV[10]<br>Mnif[11]	TAACTATGGTCATCGTTCGTCAGCAGTAACAGTAATTGCT<br>ACACCTGCTGAAACCACTGTCCCTTTTTCTTGGGCAACTC<br>TTGTTTATGTGTTGAAAGCGGAGGTCCTGAACCGGGTGT<br>TGGCTGTGCCGGACGTGGTGTAACAGTAGCTATGAAAAG<br>ACTTGAAAACTTAGGTGTTTTTGATAAGGATTTGGATGTA<br>GTCATTTATGGTGTACTTGGAGATGTTGTTTGCGGAGGTT<br>TTTCAGTGCCTTTACGTTCTCGGGCCAGGACGCCTCGGAG<br>TACCTGAGCCCCGGGCTGGTGCAGTTTGCCCGCGCCACC<br>GAGACGTACTTCAGCCTGAATAACAAGTTTAGAAACCCC<br>ACGGTGGCGCCTACGCATCTCCGGGGGTAGAGCACTGTT<br>TCGGCAAGGGGGTCATCCCGACTTACCAACCCGATGCAA<br>ACTGCGAATACCGGAGAATGTTATCACGGGAGACACACG<br>GCGGGT	AE015928<br>(4515891-<br>4515973)<br>AB019138<br>(192-363)<br>AC_000008<br>(18885-19000)<br>DQ682619<br>(847-954)	475

We analyzed each environmental sample by three separate qPCR assays targeting fecal microbe genes and by the Sketa22 assay to assess PCR inhibition.[12,13] Approximately 10% of extracts from each sample type (stored water, household entrance soil, latrine entrance soil) were analyzed in duplicate. qPCR was performed on a CFX96 Touch thermocycler (Bio-Rad, Hercules, CA, USA) with an initial 10 minute, 95 °C incubation period and cycling conditions specified by the original developers for each assay (Table S2). The 25 µL reactions consisted of 12.5 µL TaqMan Environmental Master Mix 2.0 (Applied Biosystems, Waltham, MA, USA), 2.5 µL of 10x primers/probe mix, 5 µL molecular grade water, and 5 µL DNA template. Each instrument run included three NTCs and five-point, ten-fold dilution series from three extracted PCs to construct standard curves, corresponding to triplicate reactions with $10^5 – 10^1$ or $10^6 – 10^2$ target gene copies. Samples with Sketa22 quantification cycle (Cq) values > 3 above the mean Cq of extraction controls (both NEC and PC) were considered inhibited and diluted 1:5 for further analysis.

Table S2. qPCR assay cycling conditions

assay[reference]	cycles	conditions	primer/probe (nM)	sequence (5'-3')
EC23S857[9]	40	15 s: 95 °C<br>60 s: 60 °C	F (1000)	GGTAGAGCACTGTTTtGGCA
			R (1000)	TGTCTCCCGTGATAACtTTCTC
			P (80)	6-FAM-TCATCCCGACTTACCAACCCG-BHQ1
HF183/<br>BacR287[6]	40	15 s: 95 °C<br>60 s: 60 °C	HF183 (1000)	ATCATGAGTTCACATGTCCG
			BacR287 (1000)	CTTCCTCTCAGAACCCCTATCC
			BacP234MGB (80)	6-FAM-CTAATGGAACGCATCCC-BHQplus
Mnif [11]	50	10 s: 95 °C<br>30 s: 57 °C	Mnif-202F (800)	GAAAGCGGAGGTCCTGAA
			Mnif-353R (800)	ACTGAAAAACCTCCGCAAAC
			Mnif-236P (240)	6-FAM-CCGGACGTGGTGTAACAGTAGCTA-BHQ1
Sketa22[12,13]	40	15 s: 95 °C<br>60 s: 60 °C	SketaF2 (1000)	GGTTTCCGCAGCTGGG
			SketaR22 (1000)	CCGAGCCGTCCTGGTC
			SketaP2 (80)	6-FAM-AGTCGCAGGCGGCCACCGT-BHQ1

S3 Molecular Fecal Marker Quantification

Standard curves for quantifying molecular fecal markers were fit to ten-fold dilution series of positive controls that had been spiked with reference material for all three assays and extracted alongside each batch of samples.[3] Three gBlock linear DNA fragments (Integrated DNA Technologies, Skokie, Il, USA) containing composite reference sequences for the three fecal source tracking assays (as well as additional assays considered in the associated validation study) were used as standard reference material for the positive controls (Table S1).[3,14,15] Extracting the reference material accounted for loss of target DNA during extraction but induced greater variability between dilution series constructed from different PCs. We therefore allowed both the slopes and intercepts of the standard curves to vary by qPCR instrument run and extraction batch to account for the additional variation. We fit standard curves using Bayesian multilevel regression implemented in Stan through the *brms* package.[16–18] The resulting curves were relatively linear ($R^2 \geq 95\%$) when averaged across all instrument runs and extraction batches but were somewhat inefficient, particularly for HF183 (Table S3). We estimated fecal marker concentration as the mean of the posterior predictive distribution from the corresponding standard curve for the sample-specific extraction batch, instrument run, and measured Cq.

Table S3. Mean (95% CI) estimates of standard curve parameters

**assay**	**intercept**	**slope**	**efficiency (%)**	**R^2**
EC23S857	47.9 (47.3, 48.6)	-3.50 (-3.64, -3.37)	93.1 (88.1, 98.1)	0.98 (0.97, 0.98)
HF183	47.5 (46.4, 48.6)	-3.85 (-4.07, -3.67)	81.8 (76.1, 87.4)	0.98 (0.97, 0.98)
Mnif	48.8 (47.7, 50.3)	-3.47 (-3.79, -3.23)	94.5 (83.6, 104.0)	0.95 (0.93, 0.95)

S4 Detection Limits

The limit of detection (LOD) for each assay was determined using receiver operating characteristic (ROC) analysis.[19] We expressed assay LODs in terms of the cutoff Cq—the number of amplification cycles above which the target would be considered absent from the sample. We performed ROC analysis on the local validation study data reported in Holcomb et al. (2020), considering whole-Cq increments from 10 to the maximum number of cycles specified by the assay developers as the cutoff value.[3] Diagnostic sensitivity and specificity were calculated for each cutoff value, treating any reactions with a Cq value below the cutoff as positive. The highest Cq value that maximized the Youden index, $J = sensitivity + specificity - 1$, was considered the assay LOD (Table S4).[19,20]

Sample-specific process limits of detection (PLOD) were estimated for each sample from the assay LOD Cq values, the extraction batch- and instrument run-specific standard curve estimates, and the volume of water or equivalent dry mass of soil passed through the sample filter.[21,22] For *E. coli* enumerated by culture, we assumed an assay LOD of 1 colony forming unit (cfu) per plate and calculated the PLOD for each sample as the amount of sample represented by the least-diluted plate read for that sample.[3,23] This generally corresponded to PLODs of 1 cfu/100 mL water and 100 cfu/g wet soil, with varying sample-specific PLODs for soil by dry weight after accounting for moisture content. Because PLODs varied across samples, we present the mean (standard deviation) PLODs for each assay and sample matrix in Table S4.

Table S4. qPCR assay limits of detection and mean sample-specific process limits of detection

		process limit of detection			
		soil $\log_{10}$ copies/dry g		water $\log_{10}$ copies/100 mL	
assay	assay LOD Cq	n	mean (SD)	n	mean (SD)
EC23S857	39	241	4.51 (0.12)	159	3.24 (0.13)
HF183	39	242	4.25 (0.33)	159	2.84 (0.30)
Mnif	41	241	4.18 (0.17)	158	3.02 (0.10)

S5 Laboratory Quality Control

For approximately every 10 environmental samples, we also filtered sterile phosphate buffered saline (PBS) as a laboratory blank. All PBS filters were negative for culturable *E. coli* (0/151). We included at least three NTC reactions and the NECs from the same extraction batches (typically three) as the samples on each qPCR plate. HF183 was not detected in any NTC (0/46) or NEC (0/46) reaction. Mnif was likewise absent from all NTC (0/42) and NEC (0/46) reactions. EC23S857 was detected at low concentrations in 2% of NTC (1/45) and 11% of NEC (5/46) reactions. The mean EC23S857 Cq in the negative control reactions in which it was detected was 38.1, with a minimum Cq of 37.1. These low concentrations only slightly exceeded the detection limit of 39 cycles and have been reported previously for molecular *E. coli* assays; the use of *E. coli* to produce the Environmental Master Mix is thought to occasionally result in residual *E. coli* DNA.[3,24] One soil sample had a Cq >3 above the mean Sketa22 Cq of the controls (positive and negative extraction) on the same plate and was diluted 1:5 in all further analyses to address PCR inhibition. Technical duplicate reactions were performed for a randomly selected 10% of samples of each sample type. The detection status agreed between duplicates for 94% (30/32) of EC23S857, 78% (25/32) of HF183, and 91% (29/32) of Mnif reaction pairs. We considered a sample positive if at least one of the duplicate reactions had a Cq lower than the detection limit for a given assay.

S6 Multiple Pathogen Pooled Regression Model

<u>Indices</u>

samples: $i \in 1, \dots, N$

compounds: $j \in 1, \dots, J$

pathogens: $q \in 1, \dots, Q$

covariates: $k \in 1, \dots, K$

<u>Structure</u>

$$y_{i,j,q} \sim \text{Bernoulli}\left(p_{i,j,q} \right)$$

$$\text{logit}\left(p_{i,j,q} \right) = \alpha_{samp[i]} + \alpha_{comp[j]} + \alpha_{path[q]} + \beta_{path[q]} x_i + \gamma_{1,path[q]} z_{i,1} + \cdots + \gamma_{K,path[q]} z_{i,K}$$

$$\alpha_{samp[i]} \sim \text{Normal}\left(0,\ \sigma_{samp}\right)$$

$$\alpha_{comp[j]} \sim \text{Normal}\left(0,\ \sigma_{comp}\right)$$

$$\begin{bmatrix} \alpha_{path[q]} \\ \beta_{path[q]} \\ \gamma_{1,path[q]} \\ \vdots \\ \gamma_{K,path[q]} \end{bmatrix} \sim \text{MVN}\left(\begin{bmatrix} \alpha \\ \beta \\ \gamma_1 \\ \vdots \\ \gamma_K \end{bmatrix}, \Sigma \right) \qquad \text{(S1)}$$

$$\Sigma = \tau \times \Omega \times \tau$$

$$\tau = \begin{pmatrix} \sigma_\alpha & 0 & \dots & & 0 \\ 0 & \sigma_\beta & & & \vdots \\ \vdots & & \sigma_{\gamma_1} & & \\ & & & \ddots & 0 \\ 0 & \dots & & 0 & \sigma_{\gamma_K} \end{pmatrix}$$

$$\Omega = \begin{pmatrix} 1 & \rho_{\alpha,\beta} & \cdots & \rho_{\alpha,\gamma_K} \\ \rho_{\beta,\alpha} & 1 & & \vdots \\ \vdots & & & \\ & & \ddots & \\ \rho_{\gamma_K,\alpha} & \dots & & 1 \end{pmatrix}$$

Interpretation

Table S5. Interpretation of binary pooled outcome model terms

Term	Interpretation
$y_{i,j,q}$	Detection status of pathogen q in stool i from compound j
$p_{i,j,q}$	Probability of detecting pathogen q in stool i from compound j
$\alpha_{samp[i]}$	Sample-specific random effect for sample i; the change in baseline log-odds from the overall mean for all pathogens in sample i
σ_{samp}	Group standard deviation for sample-level random effects; larger value indicates greater clustering of pathogen outcomes by sample and results in less pooling of information between samples
$\alpha_{comp[j]}$	Compound-specific random effect for compound j
σ_{comp}	Group standard deviation of compound-level random effects; larger value indicates greater clustering of pathogen outcomes by compound and results in less pooling of information between compounds
$\alpha_{path[q]}$	Pathogen-specific intercept for pathogen q; the log-odds of pathogen q for an unexposed child in the reference group for all covariates
α	Group mean of the intercept across all Q pathogens; the log-odds of a generic pathogen for an unexposed child in the reference group for all covariates
$\beta_{path[q]}$	Pathogen-specific effect of exposure on the log-odds of pathogen q, conditional on the other covariates; exp $(\beta_{path[q]})$ gives the conditional odds ratio for the effect of exposure on pathogen q
x_i	Exposure status for sample i
β	Group mean conditional exposure effect across the population of Q pathogens; the pooled estimate of the conditional effect of exposure on a generic enteric pathogen
$\gamma_{k,path[q]}$	Conditional effect of covariate k on the log-odds of pathogen q
$z_{i,k}$	Value of covariate k for sample i
γ_k	Group mean conditional effect of covariate k across the population of Q pathogens
Σ	Symmetric $K+2$ matrix with the group variance of each pathogen-varying effect (α, β, γ_k) on the diagonal and their covariances on the off-diagonals; decomposes into the scale matrix τ and the correlation matrix Ω
τ	The scale matrix for pathogen-varying effects $\alpha, \beta,\ \gamma_k$: a $K+2$ diagonal matrix of the group standard deviations $\sigma_\alpha, \sigma_\beta, \sigma_{\gamma_k}$; these standard deviations reflect the extent to which each effect varies across the group of Q pathogens, with larger values indicating greater differences between pathogens
Ω	The correlation matrix for pathogen-varying effects $\alpha, \beta,\ \gamma_k$: a $K+2$ square matrix with the pairwise correlations $\rho_{\alpha,\beta}$, etc. on the off-diagonals; these correlations reflect how the different effects co-vary by pathogen. The correlations between α and each of the other effects are of particular interest as they indicate how low or high background pathogen prevalence may modulate the exposure effect and the other covariate effects

S7 Prior Distributions

We specified prior distributions for the mixed effects logistic regression models as described in the data analysis plan for the long-term follow-up study to the MapSan trial.[25] Weakly informative prior distributions were used to regularize parameter estimates and aid computation. Because all parameters except for the correlation matrix Ω were estimated on the log-odds scale, we specified standard normal priors for all slope parameters and standard half-normal priors for all group-level standard deviation parameters:

$$\begin{aligned}
&\alpha \sim \text{Normal}(-1, 2), \qquad \sigma_\alpha \sim \text{Normal}^+(0,1) \\
&\beta \sim \text{Normal}(0,1), \qquad \sigma_\beta \sim \text{Normal}^+(0,1) \\
&\gamma_k \sim \text{Normal}(0,1), \qquad \sigma_{\gamma_k} \sim \text{Normal}^+(0,1) \\
&\sigma_{samp} \sim \text{Normal}^+(0, 1), \\
&\sigma_{comp} \sim \text{Normal}^+(0, 1) \\
&\Omega \sim \text{LKJcorr}(2)
\end{aligned} \tag{S2}$$

The $\text{Normal}(-1,2)$ prior on the overall intercept parameter α, which is on the log-odds scale, implies an expected prevalence for a generic pathogen of $\text{logit}^{-1}(-1) \approx 27\%$. This corresponds to a 68% chance that the prevalence falls between 5% and 73% and a 95% chance that the prevalence is between 0.7% and 95%.[26,27] Combined with a standard half-normal (positive constrained) hyperprior on the group standard deviation σ_α for additional variation, the pathogen-specific intercept $\alpha_{path[q]}$ for each pathogen q is capable of taking any prevalence value supported by the data with gentle regularization towards prevalence between 0% and 50%, reflecting prior knowledge of the typical frequency of individual pathogens in this setting. Such regularization can facilitate better computational behavior by softly constraining the sampler away from infeasible values to explore the parameter space more efficiently. By encouraging light smoothing, regularization can also help stabilize parameter estimates; unconstrained estimation, by contrast, can be susceptible to undue influence by ordinary noise in the

data, particularly in the context of complex, high-dimensional models and with the relatively small datasets typical of environmental microbial assessments.[27,28]

Similarly, the standard normal priors for β and γ_k on the log-odds scale imply that each covariate has a 95% chance of altering the prevalence of a pathogen with the expected value for $\alpha_{path[q]}$ (corresponding to a background prevalence of 27%) by up to 22 percentage points. Representing a large but feasible potential effect size, these priors likewise provide gentle regularization towards more moderate parameter values, centered on no effect, while allowing sufficiently strong evidence in the data to produce substantial effect estimates in either direction. In addition to assisting computationally, this mild regularization towards the null helps control false discoveries that would be expected to arise from ordinary noise in the data when comparing multiple outcomes by introducing light smoothing that requires stronger patterns in the data to register as credible effects.[29,30] The degree of smoothing applied to effect estimates for individual pathogens, $\beta_{path[q]}$ and $\gamma_{k,path[q]}$, is learned from the data and represented by the group standard deviations σ_β and σ_{γ_k}. Larger group standard deviation estimates correspond to greater variability by pathogen and thus impose relatively less smoothing on the effect estimates. The independent standard half-normal priors on the group standard deviations likewise allows for a wide range of plausible values when considered on the probability scale, while discouraging extremely large values.[26,31,32]

The group correlations between the pathogen varying effects ($\rho_{\alpha,\beta}$, ρ_{β,γ_1}, etc.) were collectively given an LKJ prior on the correlation matrix, which imposes the [-1,1] constraint on correlations but otherwise assigns approximately uniform prior density across that range when shape parameter $\eta = 1$.[28,33] Larger values of η increase the weight around zero, which can be used to regularize the correlation estimates towards smaller absolute values. We selected $\eta = 2$ to provide mild regularization for computational purposes while still permitting strong correlations when warranted.[28,34]

S8 Posterior Predicted Probabilities for Continuous Exposures

Unlike posterior predicted probabilities for binary exposure variables, where unexposed status is indicated by a value of 0 and exposed status represented by 1, the change in probability for a one-unit increase in the value of a continuous exposure variable is also dependent on the initial value of the exposure variable for each observation in the model. Simply comparing the predicted probability when each exposure value is incremented by one unit with the predicted probability for the original exposure does not account for the non-linear relationship with exposure on the probability scale. We instead estimated the instantaneous slope (i.e., first derivative) on the probability scale at the observed exposure value of each observation in the model.[35–37] We used finite differences to numerically approximate the instantaneous slope, which corresponds to the prevalence difference (PD) as the expected change in pathogen prevalence for a $\log_{10}$ increase in *E. coli* concentration.[38] For each stool sample, we predicted posterior probabilities after subtracting a small constant (5×10^{-5}) from the observed *E. coli* concentration, and again after adding 5×10^{-5} to the observed concentration. The instantaneous slope for observation i was calculated by subtracting the predicted probability for the lower concentration from the predicted probability at the higher concentration, then dividing this difference in predicted probabilities by 10^{-4} (the difference in concentration between the two prediction scenarios):

$$slope_i = \frac{P\left(x_i + \frac{\epsilon}{2}\right) - P\left(x_i - \frac{\epsilon}{2}\right)}{\epsilon}, \qquad \epsilon = 1 \times 10^{-4} \tag{S3}$$

where x_i is the observed concentration. We then estimated the marginal prevalence ratio (PR) by adding the estimated slope to the posterior predicted probability for the original observed concentration x_i before dividing by the predicted probability at the observed concentration:

$$PR_i = \frac{P(x_i) + slope_i}{p(x_i)} \tag{S4}$$

S9 Fecal Marker Occurrence by Outcome Status

The mean *E. coli* concentration and human marker detection frequency among environmental samples paired with pathogen-positive and pathogen-negative child stool are reported in Table S6 for household stored water and Table S7 for domestic soil.

Table S6. Fecal marker occurrence in household stored water samples by outcome detection status

		**Household stored water**					
		***E. coli* colonies log$_{10}$ cfu/100 mL**		***E. coli* genes log$_{10}$ copies/100 mL**		**HF183 detection**	
**Outcome**	**Status**	**n**	**mean (SD)**	**n**	**mean (SD)**	**n**	**n (%)**
Any bacteria	Negative	58	1.4 (1.4)	59	4.3 (0.7)	59	13 (22)
	Positive	127	1.4 (1.3)	127	4.2 (0.8)	127	17 (13)
C. difficile	Negative	175	1.4 (1.3)	176	4.3 (0.8)	176	28 (16)
	Positive	10	1.6 (1.6)	10	4.1 (0.8)	10	2 (20)
Campylobacter	Negative	162	1.4 (1.4)	163	4.2 (0.8)	163	24 (15)
	Positive	23	1.8 (1.2)	23	4.3 (0.6)	23	6 (26)
ETEC	Negative	136	1.4 (1.4)	137	4.2 (0.8)	137	24 (18)
	Positive	49	1.5 (1.3)	49	4.3 (0.8)	49	6 (12)
STEC	Negative	177	1.4 (1.4)	178	4.2 (0.8)	178	29 (16)
	Positive	8	1.9 (1.0)	8	4.6 (0.7)	8	1 (12)
Shigella	Negative	90	1.5 (1.4)	92	4.3 (0.7)	92	23 (25)
	Positive	95	1.3 (1.3)	94	4.2 (0.8)	94	7 (7)
Any viruses	Negative	156	1.5 (1.3)	156	4.3 (0.8)	156	24 (15)
	Positive	29	1.2 (1.5)	30	4.2 (0.8)	30	6 (20)
Norovirus	Negative	159	1.5 (1.3)	159	4.3 (0.8)	159	25 (16)
	Positive	26	1.2 (1.5)	27	4.2 (0.8)	27	5 (19)
Any protozoa	Negative	86	1.5 (1.4)	87	4.2 (0.8)	87	20 (23)
	Positive	99	1.4 (1.3)	99	4.3 (0.7)	99	10 (10)
Cryptosporidium	Negative	178	1.4 (1.4)	179	4.2 (0.8)	179	28 (16)
	Positive	7	1.8 (1.3)	7	4.6 (0.7)	7	2 (29)
Giardia	Negative	93	1.5 (1.4)	94	4.2 (0.8)	94	22 (23)
	Positive	92	1.3 (1.3)	92	4.3 (0.7)	92	8 (9)
Any STH	Negative	82	1.4 (1.4)	84	4.2 (0.8)	84	17 (20)
	Positive	81	1.5 (1.4)	79	4.3 (0.8)	79	11 (14)
Ascaris	Negative	132	1.3 (1.4)	132	4.2 (0.8)	132	22 (17)
	Positive	31	1.9 (1.2)	31	4.7 (0.4)	31	6 (19)
Trichuris	Negative	92	1.5 (1.3)	94	4.2 (0.7)	94	20 (21)
	Positive	71	1.4 (1.4)	69	4.3 (0.8)	69	8 (12)
Diarrhea	Negative	168	1.5 (1.4)	168	4.2 (0.8)	168	28 (17)
	Positive	18	1.1 (0.9)	19	4.6 (0.8)	19	2 (11)

Table S7. Fecal marker occurrence in domestic soil samples by outcome detection status

		Domestic soil							
		***E. coli* colonies log_{10} cfu/dry g**		***E. coli* genes log_{10} copies/dry g**		**HF183 detection**		**Mnif detection**	
**Outcome**	**Status**	**n**	**mean (SD)**	**n**	**mean (SD)**	**n**	**n (%)**	**n**	**n (%)**
Any bacteria	Negative	105	3.8 (1.1)	107	6.7 (1.0)	107	49 (46)	106	43 (41)
	Positive	228	3.9 (1.0)	242	6.8 (1.1)	242	116 (48)	241	114 (47)
C. difficile	Negative	320	3.8 (1.0)	330	6.8 (1.0)	330	161 (49)	328	147 (45)
	Positive	13	3.8 (1.2)	19	6.8 (1.0)	19	4 (21)	19	10 (53)
Campylobacter	Negative	291	3.9 (1.0)	307	6.8 (1.0)	307	136 (44)	305	133 (44)
	Positive	42	3.8 (1.0)	42	7.0 (1.0)	42	29 (69)	42	24 (57)
ETEC	Negative	245	3.8 (1.1)	256	6.8 (1.1)	256	119 (46)	254	110 (43)
	Positive	88	4.0 (1.0)	93	6.9 (1.0)	93	46 (49)	93	47 (51)
STEC	Negative	317	3.9 (1.0)	333	6.8 (1.1)	333	158 (47)	331	148 (45)
	Positive	16	3.8 (0.9)	16	6.8 (0.9)	16	7 (44)	16	9 (56)
Shigella	Negative	161	3.8 (1.1)	168	6.8 (1.0)	168	78 (46)	167	68 (41)
	Positive	172	3.9 (1.0)	181	6.9 (1.1)	181	87 (48)	180	89 (49)
Any viruses	Negative	280	3.9 (1.0)	296	6.8 (1.1)	296	140 (47)	295	134 (45)
	Positive	53	3.6 (1.2)	53	6.7 (1.0)	53	25 (47)	52	23 (44)
Norovirus	Negative	285	3.9 (1.0)	301	6.8 (1.1)	301	145 (48)	300	138 (46)
	Positive	48	3.6 (1.2)	48	6.6 (1.0)	48	20 (42)	47	19 (40)
Any protozoa	Negative	157	3.8 (1.1)	163	6.8 (1.1)	163	83 (51)	163	66 (40)
	Positive	176	3.9 (0.9)	186	6.9 (1.0)	186	82 (44)	184	91 (49)
Cryptosporidium	Negative	323	3.8 (1.0)	336	6.8 (1.0)	336	162 (48)	334	150 (45)
	Positive	10	4.2 (0.9)	13	6.4 (1.4)	13	3 (23)	13	7 (54)
Giardia	Negative	167	3.8 (1.1)	176	6.7 (1.1)	176	87 (49)	176	74 (42)
	Positive	166	3.9 (0.9)	173	6.9 (1.0)	173	78 (45)	171	83 (49)
Any STH	Negative	142	3.9 (1.0)	152	6.7 (1.0)	152	68 (45)	151	71 (47)
	Positive	153	3.8 (1.0)	159	6.9 (1.1)	159	78 (49)	159	71 (45)
Ascaris	Negative	231	3.8 (1.0)	247	6.7 (1.0)	247	109 (44)	246	113 (46)
	Positive	64	3.9 (1.2)	64	7.1 (1.0)	64	37 (58)	64	29 (45)
Trichuris	Negative	161	3.9 (1.0)	171	6.8 (1.0)	171	82 (48)	170	77 (45)
	Positive	134	3.8 (1.1)	140	6.9 (1.1)	140	64 (46)	140	65 (46)
Diarrhea	Negative	303	3.8 (1.0)	319	6.8 (1.0)	319	145 (45)	317	143 (45)
	Positive	32	4.3 (0.8)	32	7.3 (0.9)	32	19 (59)	32	14 (44)

S10 Marginal Prevalence Estimates

We used posterior prediction to estimate the marginal prevalence distribution of each outcome (individual pathogen, pathogen class, diarrhea) under three fecal indicator exposure scenarios: 1) the observed distribution of exposures among all participant observation-environmental sample pairs; 2) all observation-sample pairs set to unexposed; and 3) all observation-sample pairs set to exposed. Prevalence differences and prevalence ratios were calculated from the posterior prevalence predictions as described in the main text and above. The “observed” exposure scenario reflected the empirical prevalence of each outcome among the participant observation-sample pairs included in the model for a given outcome, environmental sample matrix, and fecal indicator; because the number of such pairs varied, the prevalence estimates for the observed scenario were similar but not identical to the crude prevalence for each outcome when considering only the participant observations reported in Table 2 of the main text. The “unexposed” and “exposed” scenarios were only evaluated for the human fecal indicator presence/absence for which a binary exposed/unexposed status was meaningful. Marginal PD estimates for diarrhea, individual pathogens, and pooled across all pathogens are displayed in Figure S1. The constraint at zero absolute prevalence produced truncated 95% CIs on the PD for some lower prevalence pathogens, for which the PR intervals on the relative scale were more symmetrical (Figure 1). Exposure associations with composite pathogen class variables are presented as marginal PR estimates in Figure S2 and as marginal PD estimates in Figure S3. The posterior predicted prevalence under each exposure scenario (observed, unexposed, exposed) and the corresponding marginal PR and PD estimates are reported in Table S8.

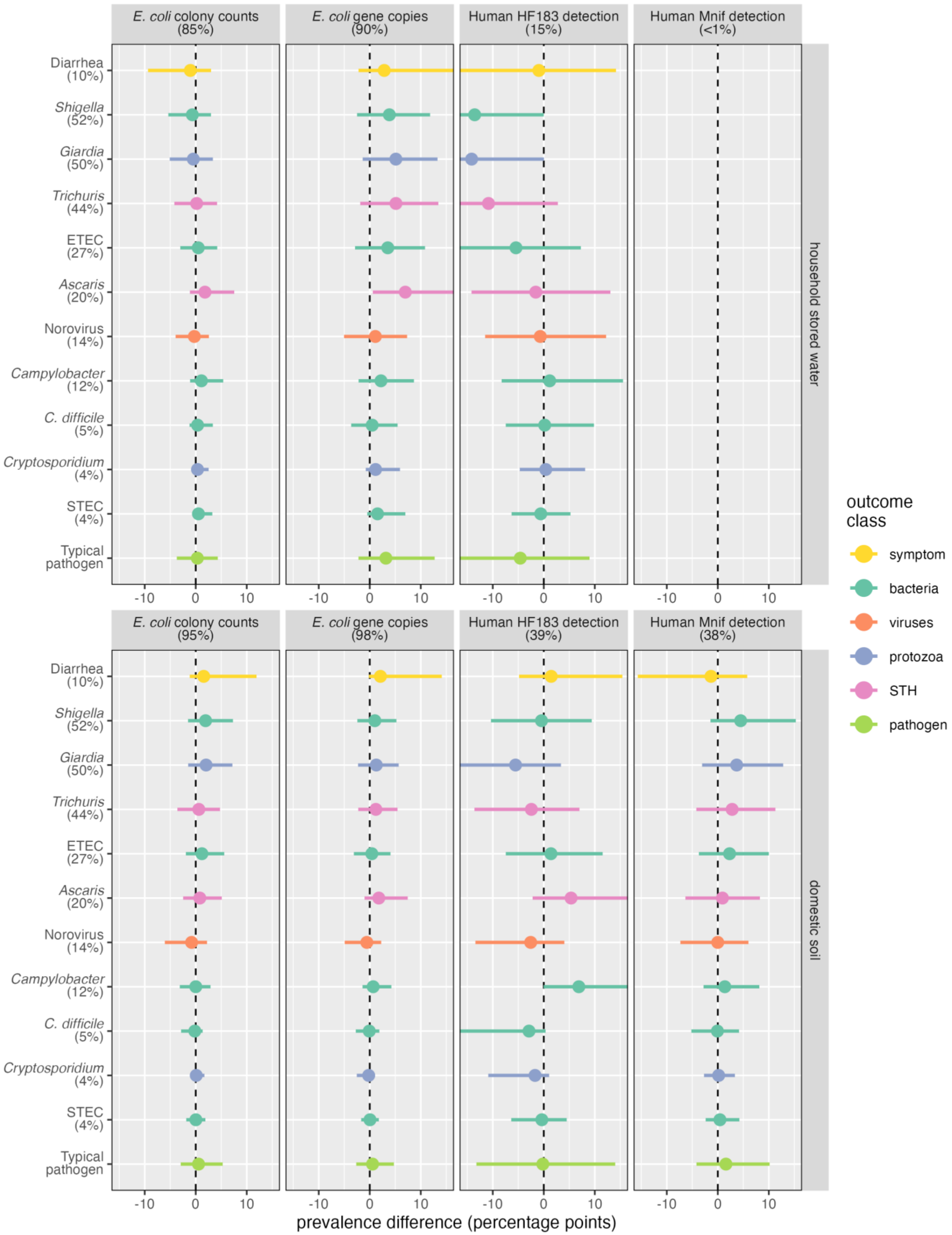

Figure S1. Mean and 95% CI marginal prevalence difference estimates of diarrhea and of enteric pathogens in stool for a ten-fold increase in E. coli concentration or detection of a human fecal marker.

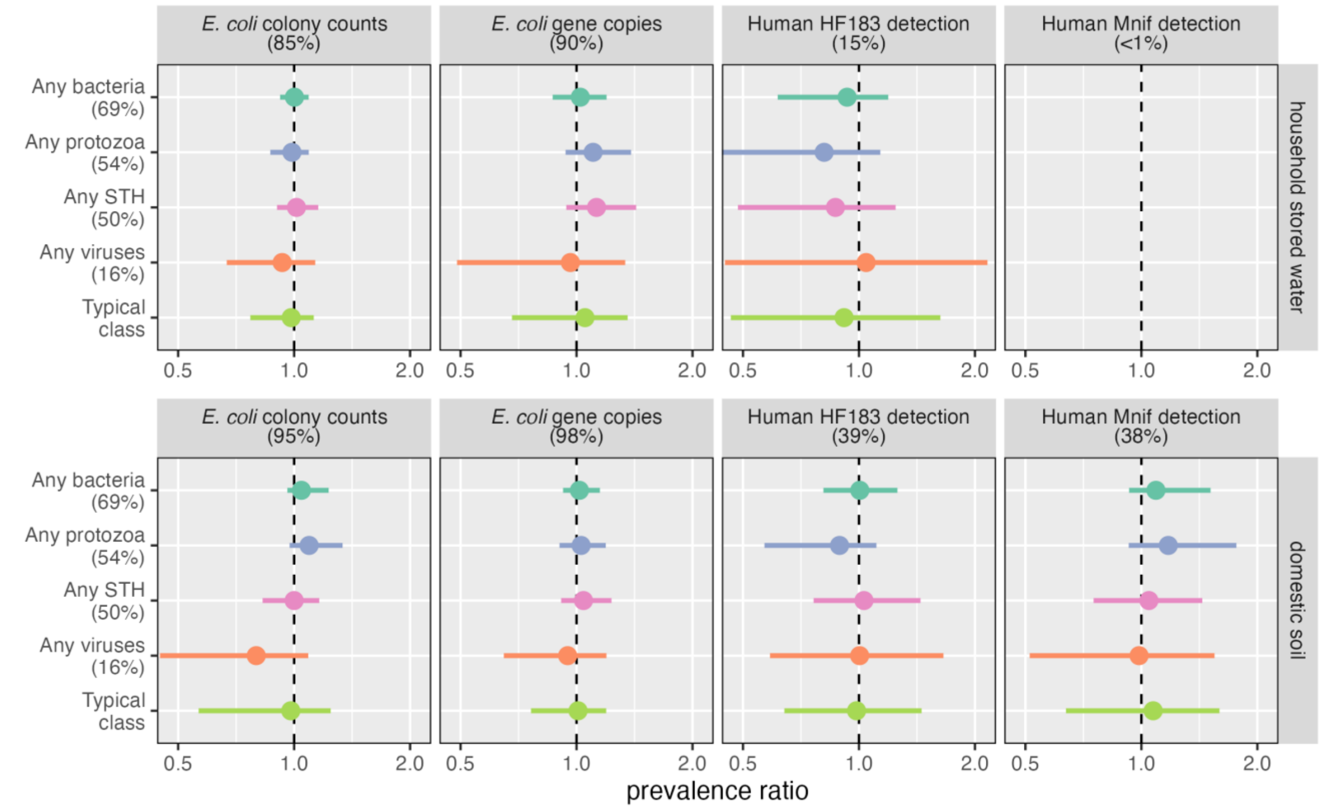

Figure S2. Mean and 95% CI marginal prevalence ratio estimates of detecting any pathogen of a given class for a ten-fold increase in *E. coli* concentration or detection of a human fecal marker.

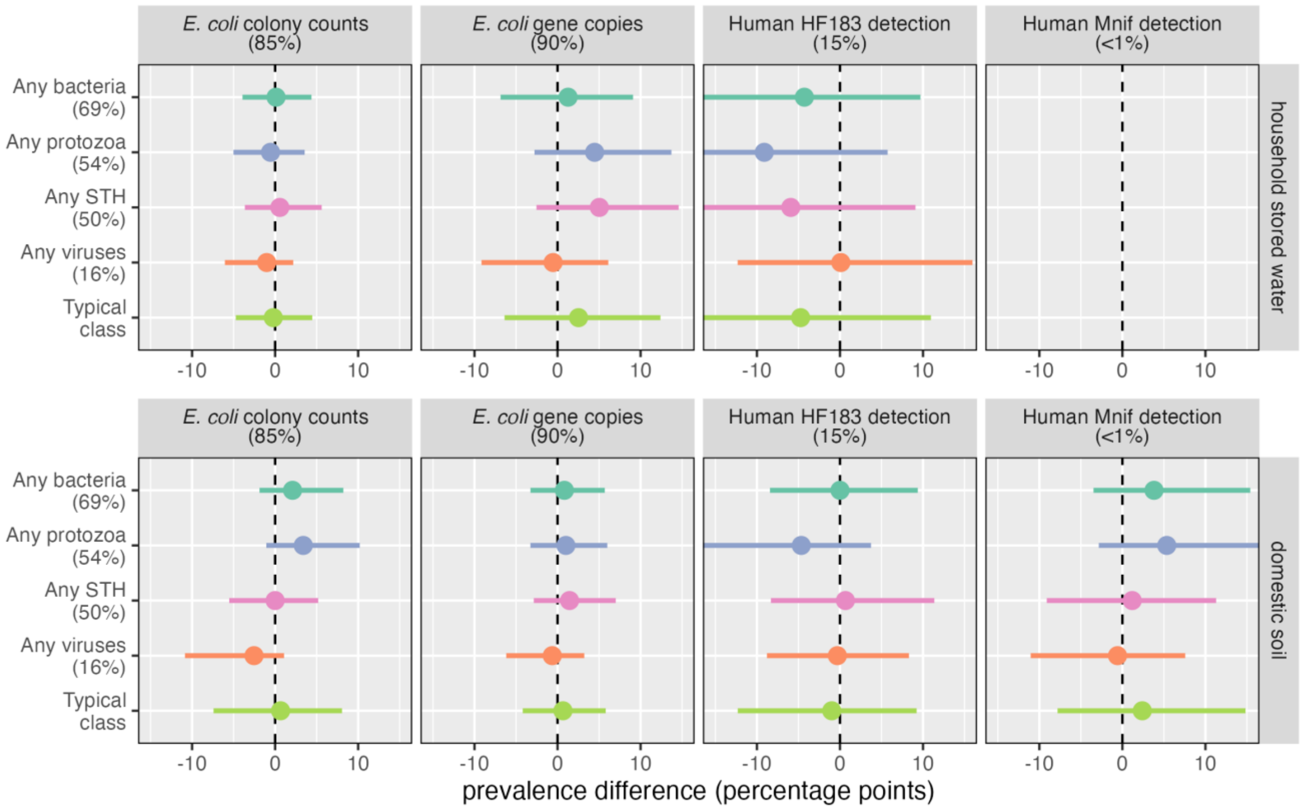

Figure S3. Mean and 95% CI marginal prevalence difference estimates of detecting any pathogen of a given class for a ten-fold increase in *E. coli* concentration or detection of a human fecal marker.

Table S8. Mean (95% CI) estimates of posterior predicted prevalence, prevalence differences, and prevalence ratios for each outcome

Outcome	Exposure	Posterior predicted prevalence[a]			PD	PR
		Observed %	Unexposed %	Exposed %	pp[b]	
Stored water samples						
Any bacteria	*E. coli* colonies	68 (25, 96)			0.1 (-3.9, 4.4)	1.0 (0.9, 1.1)
	E. coli genes	68 (25, 96)			1.3 (-6.9, 9.1)	1.0 (0.9, 1.2)
	HF183	68 (25, 96)	69 (26, 96)	65 (21, 95)	-4.3 (-20.7, 9.7)	0.9 (0.6, 1.2)
C. difficile	*E. coli* colonies	6 (0, 28)			0.4 (-1.2, 3.4)	1.1 (0.8, 1.4)
	E. coli genes	6 (0, 28)			0.5 (-3.6, 5.5)	1.1 (0.4, 1.6)
	HF183	6 (0, 28)	6 (0, 27)	6 (0, 30)	0.2 (-7.5, 9.8)	1.1 (0.4, 2.8)
Campylobacter	*E. coli* colonies	12 (2, 36)			1.1 (-1.1, 5.4)	1.1 (0.9, 1.3)
	E. coli genes	12 (2, 35)			2.2 (-2.2, 8.7)	1.2 (0.8, 1.5)
	HF183	12 (2, 35)	12 (2, 34)	13 (2, 40)	1.2 (-8.3, 15.5)	1.1 (0.5, 2.4)
ETEC	*E. coli* colonies	26 (5, 59)			0.5 (-3.0, 4.2)	1.0 (0.9, 1.2)
	E. coli genes	26 (6, 59)			3.5 (-2.9, 10.8)	1.2 (0.9, 1.4)
	HF183	26 (6, 60)	27 (6, 61)	22 (4, 55)	-5.4 (-19.8, 7.3)	0.8 (0.4, 1.3)
STEC	*E. coli* colonies	5 (0, 18)			0.5 (-0.7, 3.2)	1.1 (0.9, 1.5)
	E. coli genes	5 (0, 18)			1.5 (-0.5, 7.0)	1.3 (0.9, 1.9)
	HF183	5 (1, 17)	5 (1, 17)	5 (0, 17)	-0.6 (-6.3, 5.2)	0.9 (0.3, 2.2)
Shigella	*E. coli* colonies	51 (7, 95)			-0.7 (-5.4, 3.0)	1.0 (0.8, 1.1)
	E. coli genes	50 (7, 95)			3.8 (-2.5, 11.8)	1.1 (0.9, 1.4)
	HF183	51 (6, 95)	53 (8, 95)	39 (3, 91)	-13.5 (-35.6, 0.0)	0.7 (0.3, 1.0)
Any viruses	*E. coli* colonies	16 (2, 47)			-1.0 (-6.0, 2.2)	0.9 (0.7, 1.1)
	E. coli genes	17 (2, 46)			-0.5 (-9.2, 6.1)	1.0 (0.5, 1.3)
	HF183	17 (2, 47)	17 (2, 47)	17 (2, 50)	0.1 (-12.3, 16.0)	1.0 (0.4, 2.2)
Norovirus	*E. coli* colonies	14 (2, 40)			-0.3 (-3.9, 2.6)	1.0 (0.8, 1.2)
	E. coli genes	14 (2, 40)			1.1 (-5.0, 7.3)	1.1 (0.7, 1.4)
	HF183	14 (2, 40)	15 (2, 40)	14 (2, 42)	-0.7 (-11.5, 12.2)	1.0 (0.4, 2.0)
Any protozoa	*E. coli* colonies	53 (13, 90)			-0.6 (-5.0, 3.6)	1.0 (0.9, 1.1)
	E. coli genes	53 (13, 90)			4.5 (-2.8, 13.7)	1.1 (0.9, 1.4)
	HF183	53 (12, 91)	54 (15, 91)	45 (9, 87)	-9.1 (-28.2, 5.7)	0.8 (0.4, 1.1)
Cryptosporidium	*E. coli* colonies	4 (0, 18)			0.3 (-0.8, 2.5)	1.1 (0.8, 1.4)
	E. coli genes	4 (0, 17)			1.1 (-0.8, 5.9)	1.3 (0.8, 1.9)
	HF183	4 (0, 17)	4 (0, 17)	5 (0, 20)	0.4 (-4.7, 8.1)	1.2 (0.4, 3.1)
Giardia	*E. coli* colonies	49 (10, 89)			-0.5 (-5.1, 3.4)	1.0 (0.9, 1.1)
	E. coli genes	49 (10, 90)			5.1 (-1.4, 13.3)	1.1 (1.0, 1.4)
	HF183	49 (8, 90)	51 (11, 90)	37 (5, 83)	-14.1 (-34.5, 0.0)	0.7 (0.3, 1.0)
Any STH	*E. coli* colonies	49 (12, 87)			0.6 (-3.6, 5.6)	1.0 (0.9, 1.2)
	E. coli genes	48 (12, 86)			5.0 (-2.5, 14.6)	1.1 (0.9, 1.4)
	HF183	48 (11, 86)	49 (12, 87)	43 (9, 84)	-5.9 (-22.9, 9.1)	0.9 (0.5, 1.2)

Outcome	Exposure	Posterior predicted prevalence[a]			PD	PR
		Observed %	Unexposed %	Exposed %	pp[b]	
Ascaris	*E. coli* colonies	19 (3, 50)			1.8 (-1.1, 7.5)	1.1 (0.9, 1.4)
	E. coli genes	19 (2, 54)			7.0 (0.6, 21.2)	1.4 (1.1, 2.0)
	HF183	19 (3, 51)	20 (3, 51)	18 (3, 51)	-1.6 (-14.2, 13.1)	0.9 (0.4, 1.8)
Trichuris	*E. coli* colonies	43 (8, 84)			0.2 (-4.2, 4.2)	1.0 (0.9, 1.1)
	E. coli genes	42 (8, 83)			5.1 (-1.9, 13.4)	1.2 (0.9, 1.4)
	HF183	42 (8, 83)	43 (9, 84)	33 (5, 77)	-10.8 (-28.7, 2.8)	0.7 (0.3, 1.1)
Typical pathogen[c]	*E. coli* colonies	23 (0, 84)			0.3 (-3.7, 4.3)	1.0 (0.8, 1.3)
	E. coli genes	23 (0, 84)			3.2 (-2.2, 12.7)	1.2 (0.8, 1.7)
	HF183	23 (0, 84)	24 (0, 84)	19 (0, 74)	-4.6 (-26.6, 9.0)	0.9 (0.4, 2.1)
Diarrhea	*E. coli* colonies	10 (0, 68)			-1.1 (-9.4, 3.0)	0.8 (0.3, 1.3)
	E. coli genes	11 (0, 69)			2.8 (-2.2, 18.7)	1.4 (0.7, 2.2)
	HF183	11 (0, 67)	11 (0, 68)	10 (0, 67)	-1.0 (-18.7, 14.2)	1.0 (0.2, 3.0)
Soil samples						
Any bacteria	*E. coli* colonies	68 (10, 99)			2.1 (-1.9, 8.2)	1.0 (1.0, 1.2)
	E. coli genes	69 (11, 99)			0.8 (-3.3, 5.7)	1.0 (0.9, 1.1)
	HF183	69 (11, 99)	69 (11, 99)	69 (11, 99)	0.0 (-8.4, 9.4)	1.0 (0.8, 1.3)
	Mnif	69 (11, 99)	67 (10, 99)	71 (12, 99)	3.8 (-3.5, 15.4)	1.1 (0.9, 1.5)
C. difficile	*E. coli* colonies	4 (0, 26)			-0.2 (-2.9, 1.4)	0.9 (0.6, 1.2)
	E. coli genes	5 (0, 35)			-0.1 (-2.7, 1.9)	1.0 (0.7, 1.2)
	HF183	6 (0, 37)	7 (0, 42)	4 (0, 27)	-2.9 (-19.6, 0.4)	0.6 (0.2, 1.2)
	Mnif	6 (0, 35)	6 (0, 36)	5 (0, 35)	-0.1 (-5.2, 4.2)	1.0 (0.5, 1.6)
Campylobacter	*E. coli* colonies	13 (1, 44)			0.0 (-3.1, 2.9)	1.0 (0.8, 1.2)
	E. coli genes	12 (1, 43)			0.7 (-1.4, 4.2)	1.1 (0.9, 1.3)
	HF183	12 (0, 45)	9 (0, 35)	16 (1, 53)	6.9 (0.0, 24.4)	2.1 (1.0, 4.1)
	Mnif	12 (1, 43)	11 (1, 40)	13 (1, 44)	1.4 (-2.8, 8.1)	1.2 (0.8, 1.7)
ETEC	*E. coli* colonies	26 (2, 70)			1.2 (-1.9, 5.6)	1.1 (0.9, 1.2)
	E. coli genes	27 (2, 70)			0.4 (-3.1, 4.1)	1.0 (0.9, 1.2)
	HF183	27 (2, 70)	26 (2, 69)	27 (2, 71)	1.4 (-7.4, 11.5)	1.1 (0.7, 1.6)
	Mnif	26 (2, 70)	25 (2, 68)	28 (3, 71)	2.3 (-3.7, 10.0)	1.1 (0.9, 1.5)
STEC	*E. coli* colonies	5 (0, 23)			0.0 (-1.9, 1.9)	1.0 (0.7, 1.3)
	E. coli genes	5 (0, 22)			0.0 (-1.7, 1.8)	1.0 (0.7, 1.2)
	HF183	5 (0, 22)	5 (0, 23)	5 (0, 21)	-0.4 (-6.4, 4.5)	1.0 (0.4, 1.9)
	Mnif	5 (0, 22)	5 (0, 21)	5 (0, 23)	0.4 (-2.4, 4.2)	1.1 (0.7, 1.7)
Shigella	*E. coli* colonies	52 (3, 97)			1.9 (-1.5, 7.3)	1.1 (1.0, 1.3)
	E. coli genes	52 (3, 97)			1.0 (-2.4, 5.2)	1.0 (0.9, 1.2)
	HF183	52 (3, 97)	52 (3, 97)	51 (3, 97)	-0.4 (-10.4, 9.4)	1.0 (0.7, 1.4)
	Mnif	52 (3, 97)	50 (3, 97)	54 (4, 98)	4.5 (-1.4, 15.3)	1.2 (1.0, 1.7)

Outcome	Exposure	Posterior predicted prevalence[a]			PD	PR
		Observed %	Unexposed %	Exposed %	pp[b]	
Any viruses	*E. coli* colonies	16 (0, 65)			-2.5 (-10.9, 1.1)	0.8 (0.4, 1.1)
	E. coli genes	16 (0, 62)			-0.6 (-6.2, 3.2)	0.9 (0.6, 1.2)
	HF183	15 (0, 61)	16 (0, 61)	15 (0, 61)	-0.3 (-8.8, 8.3)	1.0 (0.6, 1.7)
	Mnif	15 (0, 60)	16 (0, 61)	15 (0, 60)	-0.6 (-11.0, 7.6)	1.0 (0.5, 1.5)
Norovirus	*E. coli* colonies	14 (1, 49)			-0.8 (-6.1, 2.2)	0.9 (0.6, 1.1)
	E. coli genes	14 (1, 47)			-0.6 (-4.9, 2.2)	1.0 (0.7, 1.1)
	HF183	14 (1, 47)	15 (1, 49)	12 (1, 44)	-2.6 (-13.4, 4.0)	0.8 (0.5, 1.3)
	Mnif	14 (1, 46)	14 (1, 46)	14 (1, 46)	0.0 (-7.3, 6.0)	1.0 (0.6, 1.5)
Any protozoa	*E. coli* colonies	53 (4, 97)			3.4 (-1.1, 10.2)	1.1 (1.0, 1.3)
	E. coli genes	53 (4, 97)			1.0 (-3.3, 6.0)	1.0 (0.9, 1.2)
	HF183	53 (4, 97)	55 (5, 97)	51 (4, 96)	-4.7 (-17.5, 3.7)	0.9 (0.6, 1.1)
	Mnif	53 (4, 97)	50 (4, 96)	56 (5, 97)	5.3 (-2.8, 17.4)	1.2 (0.9, 1.8)
Cryptosporidium	*E. coli* colonies	3 (0, 17)			0.1 (-1.1, 1.7)	1.0 (0.7, 1.3)
	E. coli genes	4 (0, 21)			-0.2 (-2.5, 1.0)	0.9 (0.6, 1.2)
	HF183	4 (0, 22)	5 (0, 24)	3 (0, 17)	-1.7 (-10.9, 1.0)	0.7 (0.2, 1.4)
	Mnif	4 (0, 21)	4 (0, 21)	4 (0, 21)	0.2 (-2.7, 3.3)	1.1 (0.6, 1.7)
Giardia	*E. coli* colonies	50 (5, 93)			2.0 (-1.5, 7.2)	1.1 (1.0, 1.2)
	E. coli genes	49 (5, 94)			1.3 (-2.3, 5.7)	1.0 (0.9, 1.2)
	HF183	49 (5, 94)	52 (5, 94)	46 (4, 93)	-5.5 (-17.1, 3.4)	0.9 (0.6, 1.1)
	Mnif	49 (4, 94)	47 (4, 93)	51 (5, 94)	3.7 (-3.1, 12.8)	1.1 (0.9, 1.5)
Any STH	*E. coli* colonies	52 (4, 96)			0.0 (-5.5, 5.2)	1.0 (0.8, 1.2)
	E. coli genes	51 (3, 96)			1.4 (-2.9, 7.0)	1.0 (0.9, 1.2)
	HF183	51 (4, 96)	51 (3, 96)	52 (4, 97)	0.7 (-8.3, 11.4)	1.0 (0.8, 1.4)
	Mnif	51 (4, 97)	51 (4, 96)	52 (4, 97)	1.2 (-9.1, 11.3)	1.0 (0.8, 1.4)
Ascaris	*E. coli* colonies	22 (1, 67)			0.8 (-2.5, 5.1)	1.0 (0.9, 1.2)
	E. coli genes	21 (1, 66)			1.8 (-1.0, 7.4)	1.1 (0.9, 1.4)
	HF183	21 (1, 66)	18 (1, 61)	24 (2, 70)	5.4 (-2.2, 19.1)	1.4 (0.9, 2.4)
	Mnif	21 (1, 66)	20 (1, 65)	21 (1, 66)	0.9 (-6.4, 8.2)	1.1 (0.7, 1.5)
Trichuris	*E. coli* colonies	45 (4, 91)			0.6 (-3.6, 4.8)	1.0 (0.9, 1.2)
	E. coli genes	45 (4, 92)			1.2 (-2.3, 5.4)	1.0 (0.9, 1.2)
	HF183	45 (4, 92)	46 (4, 92)	43 (4, 91)	-2.4 (-13.6, 7.0)	0.9 (0.6, 1.3)
	Mnif	45 (4, 92)	44 (4, 91)	46 (4, 93)	2.8 (-4.2, 11.3)	1.1 (0.9, 1.5)
Typical pathogen	*E. coli* colonies	23 (0, 88)			0.6 (-2.9, 5.3)	1.0 (0.7, 1.2)
	E. coli genes	24 (0, 89)			0.5 (-2.6, 4.7)	1.0 (0.8, 1.2)
	HF183	24 (0, 89)	24 (0, 90)	23 (0, 88)	-0.2 (-13.2, 14.0)	1.0 (0.3, 2.6)
	Mnif	23 (0, 89)	22 (0, 87)	24 (0, 90)	1.6 (-4.2, 10.2)	1.1 (0.7, 1.6)

Outcome	Exposure	Posterior predicted prevalence[a]			PD	PR
		Observed %	Unexposed %	Exposed %	pp[b]	
Diarrhea	*E. coli* colonies	10 (0, 75)			1.6 (-1.2, 11.9)	1.3 (0.8, 1.8)
	E. coli genes	9 (0, 74)			2.1 (-0.2, 14.1)	1.4 (0.9, 1.9)
	HF183	9 (0, 74)	9 (0, 70)	10 (0, 76)	1.5 (-4.8, 15.4)	1.4 (0.6, 3.2)
	Mnif	9 (0, 74)	10 (0, 77)	9 (0, 71)	-1.3 (-15.7, 5.8)	0.9 (0.3, 2.0)

[a] Predicted population-level prevalence of outcome assuming: 1) the observed exposure values; 2) the entire population was unexposed; and 3) the entire population was exposed. Unexposed and exposed predicted prevalence only apply to the binary exposures assessed for human fecal markers

[b] pp: percentage points

[c] Pooled exposure effect across all the pathogens included in the model; predicted using the group means and standard deviations estimated for the pathogen-varying slopes and intercepts

S11 Supplementary References

(1) Knee, J.; Sumner, T.; Adriano, Z.; Anderson, C.; Bush, F.; Capone, D.; et al. Effects of an Urban Sanitation Intervention on Childhood Enteric Infection and Diarrhea in Maputo, Mozambique: A Controlled before-and-after Trial. *eLife* **2021**, *10*, e62278. https://doi.org/10.7554/elife.62278.

(2) Cox, A. M.; Goodwin, K. D. Sample Preparation Methods for Quantitative Detection of DNA by Molecular Assays and Marine Biosensors. *Mar Pollut Bull* **2013**, *73* (1), 47–56. https://doi.org/10.1016/j.marpolbul.2013.06.006.

(3) Holcomb, D. A.; Knee, J.; Sumner, T.; Adriano, Z.; De Bruijn, E.; Nalá, R.; et al. Human Fecal Contamination of Water, Soil, and Surfaces in Households Sharing Poor-Quality Sanitation Facilities in Maputo, Mozambique. *Int J Hyg Environ Health* **2020**, *226*, 113496. https://doi.org/10.1016/j.ijheh.2020.113496.

(4) Haugland, R. A.; Siefring, S.; Lavender, J.; Varma, M. Influences of Sample Interference and Interference Controls on Quantification of Enterococci Fecal Indicator Bacteria in Surface Water Samples by the qPCR Method. *Water Res* **2012**, *46* (18), 5989–6001. https://doi.org/10.1016/j.watres.2012.08.017.

(5) Kildare, B. J.; Leutenegger, C. M.; McSwain, B. S.; Bambic, D. G.; Rajal, V. B.; Wuertz, S. 16S rRNA-Based Assays for Quantitative Detection of Universal, Human-, Cow-, and Dog-Specific Fecal Bacteroidales: A Bayesian Approach. *Water Res* **2007**, *41* (16), 3701–3715. https://doi.org/10.1016/j.watres.2007.06.037.

(6) Green, H. C.; Haugland, R. A.; Varma, M.; Millen, H. T.; Borchardt, M. A.; Field, K. G.; et al. Improved HF183 Quantitative Real-Time PCR Assay for Characterization of Human Fecal Pollution in Ambient Surface Water Samples. *Appl Environ Microbiol* **2014**, *80* (10), 3086–3094. https://doi.org/10.1128/aem.04137-13.

(7) Green, H. C.; Dick, L. K.; Gilpin, B.; Samadpour, M.; Field, K. G. Genetic Markers for Rapid PCR-Based Identification of Gull, Canada Goose, Duck, and Chicken Fecal Contamination in Water. *Appl Environ Microbiol* **2012**, *78* (2), 503–510. https://doi.org/10.1128/AEM.05734-11.

(8) Weidhaas, J. L.; Macbeth, T. W.; Olsen, R. L.; Sadowsky, M. J.; Norat, D.; Harwood, V. J. Identification of a *Brevibacterium* Marker Gene Specific to Poultry Litter and Development of a Quantitative PCR Assay. *J Appl Microbiol* **2010**, *109* (1), 334–347. https://doi.org/10.1111/j.1365-2672.2010.04666.x.

(9) Chern, E. C.; Siefring, S.; Paar, J.; Doolittle, M.; Haugland, R. A. Comparison of Quantitative PCR Assays for *Escherichia Coli* Targeting Ribosomal RNA and Single Copy Genes. *Lett Appl Microbiol* **2011**, *52* (3), 298–306. https://doi.org/10.1111/j.1472-765X.2010.03001.x.

(10) Jothikumar, N.; Cromeans, T. L.; Hill, V. R.; Lu, X.; Sobsey, M. D.; Erdman, D. D. Quantitative Real-Time PCR Assays for Detection of Human Adenoviruses and Identification of Serotypes 40 and 41. *Appl Environ Microbiol* **2005**, *71* (6), 3131–3136. https://doi.org/10.1128/AEM.71.6.3131-3136.2005.

(11) Johnston, C.; Ufnar, J. A.; Griffith, J. F.; Gooch, J. A.; Stewart, J. R. A Real-Time qPCR Assay for the Detection of the *nifH* Gene of *Methanobrevibacter Smithii*, a Potential Indicator of Sewage Pollution. *J Appl Microbiol* **2010**, *109* (6), 1946–1956. https://doi.org/10.1111/j.1365-2672.2010.04824.x.

(12) Haugland, R. A.; Siefring, S. C.; Wymer, L. J.; Brenner, K. P.; Dufour, A. P. Comparison of Enterococcus Measurements in Freshwater at Two Recreational Beaches by Quantitative

Polymerase Chain Reaction and Membrane Filter Culture Analysis. *Water Res* **2005**, *39* (4), 559–568. https://doi.org/10.1016/j.watres.2004.11.011.

(13) Haugland, R. A.; Varma, M.; Sivaganesan, M.; Kelty, C.; Peed, L.; Shanks, O. C. Evaluation of Genetic Markers from the 16S rRNA Gene V2 Region for Use in Quantitative Detection of Selected Bacteroidales Species and Human Fecal Waste by qPCR. *Syst Appl Microbiol* **2010**, *33* (6), 348–357. https://doi.org/10.1016/j.syapm.2010.06.001.

(14) Kodani, M.; Winchell, J. M. Engineered Combined-Positive-Control Template for Real-Time Reverse Transcription-PCR in Multiple-Pathogen-Detection Assays. *J Clin Microbiol* **2012**, *50* (3), 1057–1060. https://doi.org/10.1128/jcm.05987-11.

(15) Liu, J.; Gratz, J.; Amour, C.; Kibiki, G.; Becker, S.; Janaki, L.; et al. A Laboratory-Developed TaqMan Array Card for Simultaneous Detection of 19 Enteropathogens. *J Clin Microbiol* **2013**, *51* (2), 472–480. https://doi.org/10.1128/JCM.02658-12.

(16) Sivaganesan, M.; Haugland, R. A.; Chern, E. C.; Shanks, O. C. Improved Strategies and Optimization of Calibration Models for Real-Time PCR Absolute Quantification. *Water Res* **2010**, *44* (16), 4726–4735. https://doi.org/10.1016/j.watres.2010.07.066.

(17) Stan Development Team. *RStan: The R Interface to Stan*; version 2.21.1; 2020. http://mc-stan.org/.

(18) Bürkner, P.-C. Brms: An R Package for Bayesian Multilevel Models Using Stan. *J Stat Softw* **2017**, *80* (1). https://doi.org/10.18637/jss.v080.i01.

(19) Nutz, S.; Döll, K.; Karlovsky, P. Determination of the LOQ in Real-Time PCR by Receiver Operating Characteristic Curve Analysis: Application to qPCR Assays for Fusarium Verticillioides and F. Proliferatum. *Anal Bioanal Chem* **2011**, *401* (2), 717–726. https://doi.org/10.1007/s00216-011-5089-x.

(20) Fluss, R.; Faraggi, D.; Reiser, B. Estimation of the Youden Index and Its Associated Cutoff Point. *Biom J* **2005**, *47* (4), 458–472. https://doi.org/10.1002/bimj.200410135.

(21) Verbyla, M. E.; Symonds, E. M.; Kafle, R. C.; Cairns, M. R.; Iriarte, M.; Mercado Guzmán, A.; et al. Managing Microbial Risks from Indirect Wastewater Reuse for Irrigation in Urbanizing Watersheds. *Environ Sci Technol* **2016**, *50* (13), 6803–6813. https://doi.org/10.1021/acs.est.5b05398.

(22) Ahmed, W.; Bivins, A.; Metcalfe, S.; Smith, W. J. M.; Verbyla, M. E.; Symonds, E. M.; et al. Evaluation of Process Limits of Detection and Quantification Variation of SARS-CoV-2 RT-qPCR and RT-dPCR Assays for Wastewater Surveillance. *Water Res* **2022**, *213*, 118132. https://doi.org/10.1016/j.watres.2022.118132.

(23) Levy, K.; Nelson, K. L.; Hubbard, A.; Eisenberg, J. N. S. Rethinking Indicators of Microbial Drinking Water Quality for Health Studies in Tropical Developing Countries: Case Study in Northern Coastal Ecuador. *Am Soc Trop Med Hyg* **2012**, *86* (3), 499–507. https://doi.org/10.4269/ajtmh.2012.11-0263.

(24) Shrestha, A.; Dorevitch, S. Evaluation of Rapid qPCR Method for Quantification of *E. Coli* at Non-Point Source Impacted Lake Michigan Beaches. *Water Res* **2019**, *156*, 395–403. https://doi.org/10.1016/j.watres.2019.03.034.

(25) Holcomb, D.; Capone, C.; Cumbane, V.; Knee, J.; Kowaslky, E.; Lai, A.; et al. *Data Analysis Plan: Long-Term Impact of an Urban Sanitation Intervention on Child Health in Low-Income Neighborhoods of Maputo City, Mozambique: A Cross-Sectional Follow-up Five Years Post-*

Intervention in the Maputo Sanitation (MapSan) Trial. Version 4 (Revised 2023-01-20).; 2023. https://osf.io/e7pvk/.

(26) Holcomb, D. A.; Knee, J.; Capone, D.; Sumner, T.; Adriano, Z.; Nalá, R.; et al. Impacts of an Urban Sanitation Intervention on Fecal Indicators and the Prevalence of Human Fecal Contamination in Mozambique. *Environ Sci Technol* **2021**, *55* (17), 11667–11679. https://doi.org/10.1021/acs.est.1c01538.

(27) Gelman, A.; Carpenter, B. Bayesian Analysis of Tests with Unknown Specificity and Sensitivity. *J R Stat Soc C: Appl Stat* **2020**, *69* (5), 1269–1283. https://doi.org/10.1111/rssc.12435.

(28) McElreath, R. *Statistical Rethinking: A Bayesian Course with Examples in R and Stan*, 2nd ed.; Chapman & Hall/CRC Texts in Statistical Science; CRC Press: Boca Raton, 2020.

(29) Gelman, A.; Hill, J.; Yajima, M. Why We (Usually) Don't Have to Worry About Multiple Comparisons. *J Res Educ Eff* **2012**, *5* (2), 189–211. https://doi.org/10.1080/19345747.2011.618213.

(30) Mitchell, S.; Gelman, A.; Ross, R.; Chen, J.; Bari, S.; Huynh, U. K.; et al. The Millennium Villages Project: A Retrospective, Observational, Endline Evaluation. *Lancet Glob Health* **2018**, *6* (5), e500–e513. https://doi.org/10.1016/s2214-109x(18)30065-2.

(31) Gelman, A. Prior Distribution for Variance Parameters in Hierarchical Models. *Bayesian Anal* **2006**, *1* (3), 515–533. https://doi.org/10.1214/06-ba117a.

(32) Stan Development Team. *Prior Choice Recommendations*; 2023. https://github.com/stan-dev/stan/wiki/Prior-Choice-Recommendations (accessed 2023-11-17).

(33) Lewandowski, D.; Kurowicka, D.; Joe, H. Generating Random Correlation Matrices Based on Vines and Extended Onion Method. *J Multivariate Anal* **2009**, *100* (9), 1989–2001. https://doi.org/10.1016/j.jmva.2009.04.008.

(34) Stan Development Team. *Stan User's Guide*; version 2.33; 2023. https://mc-stan.org/docs/stan-users-guide/index.html.

(35) Arel-Bundock, V. 4. Slopes. In *The Marginal Effects Zoo*; 2023. http://web.archive.org/web/20231112072026/https://marginaleffects.com/articles/slopes.html#definition.

(36) Heiss, A. *Marginalia: A Guide to Figuring out What the Heck Marginal Effects, Marginal Slopes, Average Marginal Effects, Marginal Effects at the Mean, and All These Other Marginal Things Are*; 2022. https://doi.org/10.59350/40xaj-4e562 (accessed 2023-09-15).

(37) Arel-Bundock, V.; Greifer, N.; Heiss, A. How to Interpret Statistical Models Using Marginaleffects in R and Python. *J Stat Softw* **2024**, *111* (9). https://doi.org/10.18637/jss.v111.i09.

(38) Simpson, G. L. Modelling Palaeoecological Time Series Using Generalised Additive Models. *Front Ecol Evol* **2018**, *6*, 149. https://doi.org/10.3389/fevo.2018.00149.
